# Evidence of vitamin D synthesis in insects exposed to UVb light

**DOI:** 10.1038/s41598-018-29232-w

**Published:** 2018-07-17

**Authors:** D. G. A. B. Oonincx, P. van Keulen, M. D. Finke, F. M. Baines, M. Vermeulen, G. Bosch

**Affiliations:** 1 Laboratory of Entomology, Department of Plant Sciences, Wageningen University & Research, P.O. Box 16, 6700 AA Wageningen, The Netherlands; 2 Animal Nutrition Group, Department of Animal Sciences, Wageningen University & Research, P.O. Box 338, 6700 AH Wageningen, The Netherlands; 3 Mark Finke LLC, 17028 E Wildcat Dr, Rio Verde, AZ 85263 USA; 4 UV Guide UK, Greenfield, School Lane, Govilon, Abergavenny, NP7 9NT Wales UK; 5 TNO Triskelion, Nutrient Analysis team, Utrechtseweg 48, Zeist, The Netherlands; 6 Present Address: CCIC Europe Food Test, Lelystad, The Netherlands

## Abstract

Vertebrates obtain the prohormone vitamin D primarily by endogenous cutaneous synthesis under ultraviolet b (UVb) exposure. To date, endogenous synthesis of vitamin D in insects has never been investigated. In an initial experiment, we exposed four insect species which differ in ecology and morphology (migratory locusts, house crickets, yellow mealworms and black soldier fly larvae (BSFL)) to a low irradiance UVb source. In a second experiment we exposed these species to a higher UV irradiance, and in a third we tested the effect of exposure duration on vitamin D concentrations in yellow mealworms. Low irradiance UVb tended to increase vitamin D_3_ levels in house crickets, vitamin D_2_ levels in BSFL and vitamin D_2_ and D_3_ in yellow mealworms. Higher UVb irradiance increased vitamin D_3_ levels in all species but BSFL. Both BSFL and migratory locusts had increased vitamin D_2_ levels. Longer UVb exposure of yellow mealworms increased vitamin D_2_ and increased vitamin D_3_ until a plateau was reached at 6400 IU/kg. This study shows that insects can synthesize vitamin D *de novo* and that the amounts depend on UVb irradiance and exposure duration.

## Introduction

Vitamin D metabolites perform a hormonal function in a wide variety of animal species^1–3^. These animals can obtain vitamin D either by oral ingestion or via *de novo* synthesis^4,5^. *De novo* synthesis requires exposure of a vitamin D precursor to ultraviolet light with a wavelength between 280 and 320 nm (UVb), followed by a temperature dependent step^6,7^. Humans, birds, reptiles, amphibians and fish can use both strategies, although *de novo* synthesis seems the primary route to acquire a sufficient vitamin D status^8–11^. Ergosterol is the primary vitamin D precursor in plants, yeasts and fungi, which UVb light converts to vitamin D_2_, whereas 7-dehydrocholesterol (7DHC) is the precursor which forms vitamin D_3_ in vertebrates^12,13^. The vertebrate liver hydroxylates either form of vitamin D to 25-hydroxycholecalciferol (25(OH)D) which is the major circulating form of vitamin D^14^. This can be further hydroxylated to 1,25-dihydroxycholecalciferol (1,25(OH)_2_D) which is the hormonally most active form of vitamin D^14^. This form is best known as an endocrine regulator of calcium and phosphorus metabolism in vertebrates^15^. However, it also serves autocrine and paracrine functions in cellular proliferation, differentiation, and apoptosis, as well as in the innate immune system^16–21^.

Whereas the importance of vitamin D in vertebrate physiology is well documented, little is known for invertebrates. Moreover, in the largest animal class, the insects, its metabolism has not been studied. Vitamin D has been detected in various insect species (Table 1) although its physiological role is unknown. Concentrations differ greatly between species. High concentrations of vitamin D (538 and 1444 IU/kg fresh insect) are reported for wild specimens of two species collected in the field, where they can be exposed to solar UVb radiation^22,23^. Most commercially produced insects have far lower vitamin D concentrations^9,24–26^. As these are normally not exposed to sunlight or other sources of UVb radiation, they likely obtain their vitamin D solely via the diet. Whether insects are capable of *de novo* synthesis is hitherto unknown. Furthermore, this animal class is highly diverse and contains species with large differences in their morphology and ecology, including their degree of exposure to solar radiation. These aspects could affect their vitamin D synthesizing capacity and hence their vitamin D content. Therefore, we conducted three experiments in which we investigated: 1) whether insect species, differing in phylogeny and ecology have the capacity to synthesize vitamin D when exposed to UVb, 2) whether exposure to a higher UVb irradiance leads to a higher vitamin D concentration, and 3) how the duration of UVb exposure affects the vitamin D concentration.

**Table 1:** Literature data for vitamin D_3_ content (IU/kg fresh matter) of insects.

Species	Vitamin D_3_	Life stage	Origin	Reference
Pallid-winged grasshoppers	102	Adults	Wild	^22^
Rhinoceros beetles	538	Adults	Wild	^22^
White lined sphinx moth	<80	Adults	Wild	^22^
Escamoles ants	1444	Pupae	Wild	^23^
Superworms	<LOD	Larvae	Produced	^24^^*,^^25^^**^
Giant mealworm	<LOD	Larvae	Produced	^24^^*^
Yellow mealworms	<LOD	Larvae	Produced	^24^^*,^^25^^**^
Yellow mealworms	<LOD	Adults	Produced	^24^^*^
Waxworms	<LOD	Larvae	Produced	^24^^*,^^25^^**^
Silkworms	<LOD	Larvae	Produced	^24^^*^
Cricket	<LOD	Adult	Produced	^24^^*,^^25^^**^
Cricket	<LOD	Nymph	Produced	^24^^*^
Desert locusts	61	Nymphs	Produced	^9^^***^
Desert locusts	95	Adults	Produced	^9^^***^
Migratory locusts	33	Nymphs	Produced	^9^^***^
Migratory locusts	64	Adults	Produced	^9^^***^
House crickets	280	Adults	Produced	^9^^***^
Yellow mealworms	45	Larvae	Produced	^9^^***^
Black soldier fly	100	Larvae	Produced	^26^
Tebo worms	159	Larvae	Produced	^26^
Turkestan cockroaches	193	Nymphs	Produced	^26^
House flies	<20	Adults	Produced	^26^

*Limit of detection was 256 IU/kg.

**Limit of detection was 40 IU/kg.

***Values recalculated assuming a dry matter content of 30%.

## Results

None of the diet ingredients provided to the insect species contained detectable levels of vitamin D_2_ (Table 2). However, the complete chicken feed provided to the house crickets and BSFL in the first experiment had a high concentration of vitamin D_3_ (12,000 IU/kg DM). Therefore, commercial cricket feed with a lower concentration (580 IU/kg DM) was used for these species in the second experiment.

**Table 2:** Vitamin D_2_ and D_3_ concentrations (IU/kg) in diets (as fed) provided to four insect species and in these species (fresh matter) at the start of experiment 1 and 2.

Diet ingredients	Vitamin D_2_	Vitamin D_3_
Carrot (*Daucus carota*)	<20	<10
Ryegrass (*Lolium perenne*)	<20	<10
Wheat bran	<20	<10
Mealworm feed (experiment 1)	<20	590
Mealworm feed (experiment 2)	<20	140
Chicken feed (experiment 1)	<20	12000
Cricket feed (experiment 2)	<20	580
Migratory locusts	<20	<10
House crickets	<20	19
Yellow mealworms	<20	55
Black soldier fly larvae	245	420

**Figure 1:**
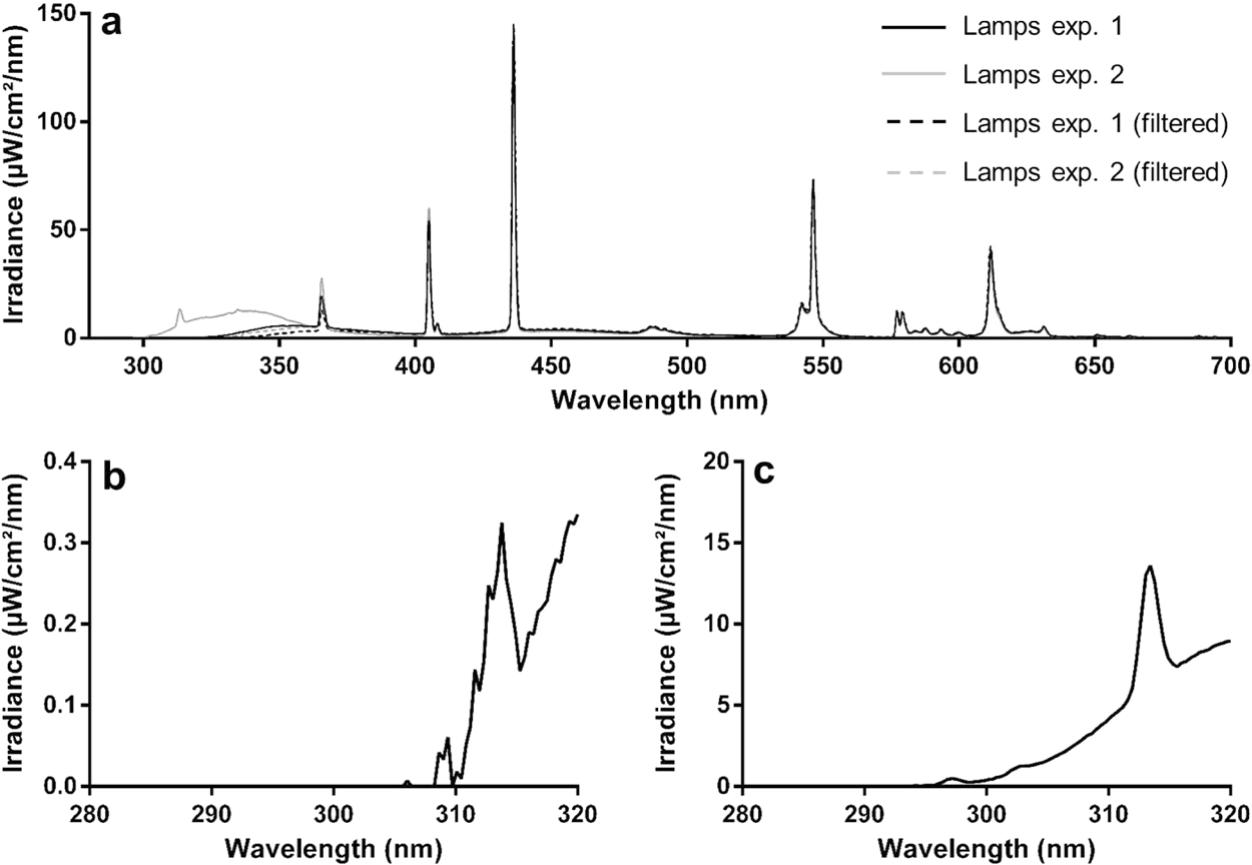
Spectral power distribution of the lamps used in experiment 1 and 2, with and without a UVb filter (**a**) and the UVb range of lamp 1 (**b**) and lamp 2 (**c**).

The spectral power distribution of the lamps used in the first and second experiment was similar (Fig. 1a) except in the UVa and UVb region (Fig. 1a and b). In the control treatments UVb radiation was completely blocked and UVa radiation was reduced by 44% in the first, and by 65% in the second experiment due to the glass filter. This glass filter also led to slightly lower temperatures (~1 °C; Tables 3 and 4) in the unexposed treatment.

**Table 3:** Environmental conditions (UVb irradiance, UV index and temperature) of four insect species exposed or not exposed (Control) to UVb radiation during the first experiment.

Species	Migratory locusts		House crickets		Yellow mealworms		Black soldier fly	
	Control	Exposed	Control	Exposed	Control	Exposed	Control	Exposed
UVb irradiance (µW/cm^2^)	0.0 ± 0.00	24.2 ± 1.56**	0.0 ± 0.00	17.4 ± 1.38**	0.0 ± 0.00	14.2 ± 1.32**	0.0 ± 0.00	26.8 ± 1.59**
UV index	0.0 ± 0.00	0.7 ± 0.11**	0.0 ± 0.00	0.6 ± 0.10**	0.0 ± 0.00	0.5 ± 0.11**	0.0 ± 0.00	0.8 ± 0.12**
Temperature (°C)	34.7 ± 0.38	35.6 ± 0.33**	27.8 ± 0.56	28.7 ± 0.57**	30.4 ± 1.56	31.3 ± 1.49**	26.3 ± 0.65	28.4 ± 0.69**

All data are reported as mean ± SD.

**P ≤ 0.001.

**Table 4:** Environmental conditions (UVb irradiance, UV index and temperature) of four insect species exposed or not exposed (Control) to UVb radiation during the second experiment.

Species	Migratory locusts		House crickets		Yellow mealworms		Black soldier fly	
	Control	Exposed	Control	Exposed	Control	Exposed	Control	Exposed
UVb irradiance (µW/cm^2^)	0.0 ± 0.00	90.0 ± 5.54**	0.0 ± 0.00	73.7 ± 4.36**	0.0 ± 0.00	70.1 ± 3.10**	0.0 ± 0.00	80.3 ± 2.05**
UV index	0.0 ± 0.00	4.6 ± 0.53**	0.0 ± 0.00	3.5 ± 0.28**	0.0 ± 0.00	3.3 ± 0.23**	0.0 ± 0.00	3.8 ± 0.22**
Temperature (°C)	34.6 ± 0.89	35.5 ± 0.63**	28.1 ± 0.50	28.8 ± 0.69**	30.4 ± 0.65	31.8 ± 0.55**	30.3 ± 0.37	30.8 ± 0.59**

All data are reported as mean ± SD.

**P ≤ 0.001.

**Figure 2:**
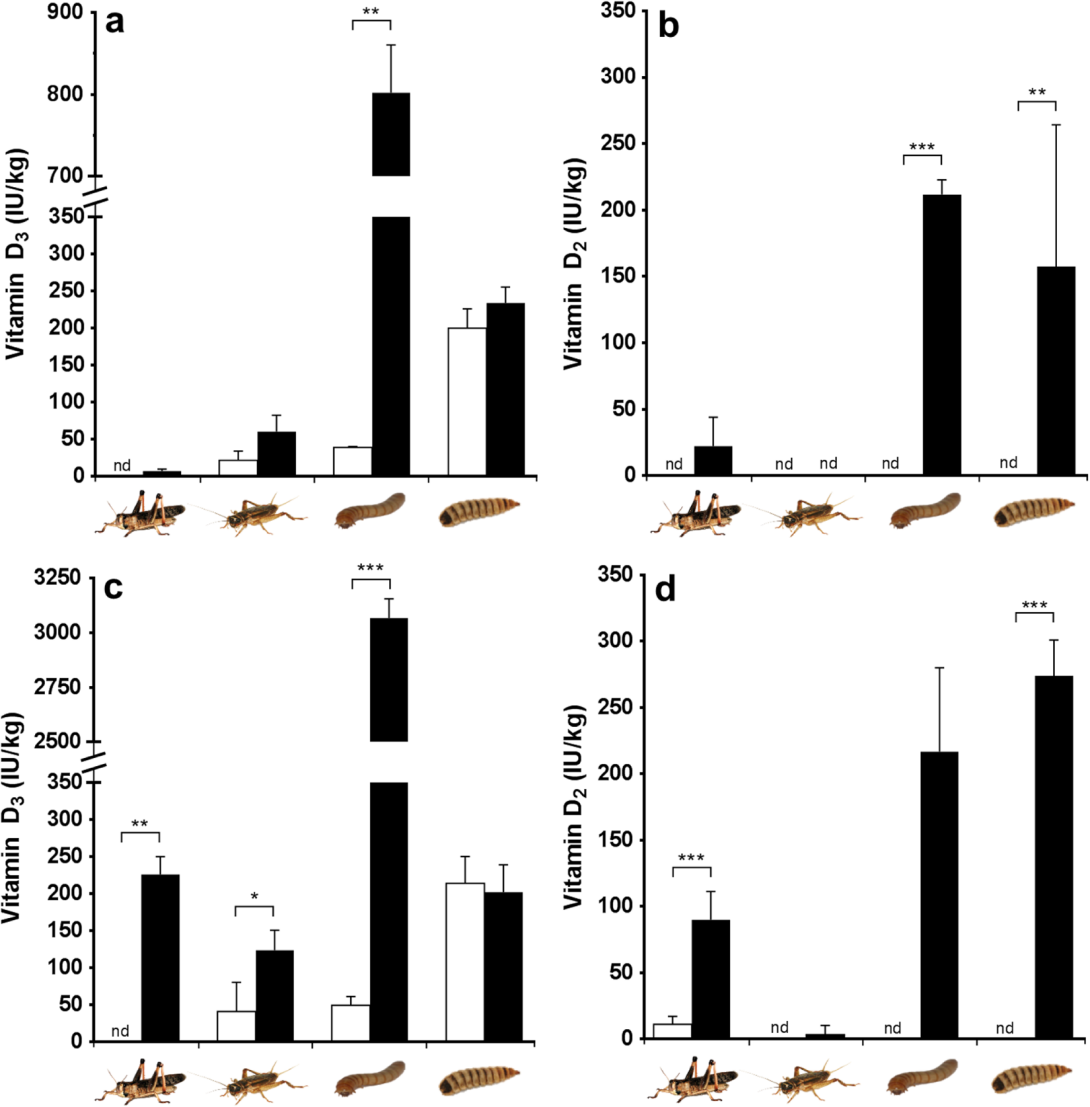
Vitamin D_3_ (**a** and **c**) and vitamin D_2_ (panel **b** and **d**) concentrations (IU/kg of fresh matter) of non-exposed (□) and UVb exposed (■) migratory locusts (*Locusta migratoria*), house crickets (*Acheta domesticus*), yellow mealworms (*Tenebrio molitor*) and black soldier fly larvae (*Hermetia illucens*) at the end of the first (**a**,**b**) and second (**c**,**d**) experiment. nd = not detected (<10 IU/kg); *P ≤ 0.05, **P ≤ 0.01, ***P ≤ 0.001; values are means ± SD. Photos by Mandy Decker.

In the first experiment UVb exposure greatly increased vitamin D_3_ concentrations (39 vs. 802 IU/kg; P = 0.008) in the substrate-dwelling yellow mealworms (Fig. 2a). The vitamin D_3_ concentrations in BSFL, also a substrate dwelling species, were not affected by UVb exposure (P = 0.345). For the above-ground living species, the UVb exposure had no effect on migratory locusts (P = 0.310), whereas in house crickets vitamin D_3_ concentrations tended (P = 0.056) to increase. Vitamin D_2_ concentrations were low or non-detectable and not affected by exposure in the migratory locusts (P = 0.151) and house crickets (P = 1.000), whereas these were higher in UVb exposed yellow mealworms (P < 0.001) and BSFL (P = 0.008) (Fig. 2b).

In the second experiment, UVb exposure elevated vitamin D_3_ concentrations in migratory locusts (0 vs. 226 IU/kg; P = 0.008), house crickets (41 vs. 124 IU/kg; P = 0.032) and yellow mealworms (50 vs. 3067 IU/kg; P < 0.001) whereas those in BSFL were unaffected (P = 0.548) (Fig. 2c). Vitamin D_2_ concentrations increased in UVb-exposed BSFL (P = 0.008) and migratory locusts (P = 0.008) and tended to be higher in UVb-exposed yellow mealworms (P = 0.100) (Fig. 2d). UVb exposure did not affect vitamin D_2_ concentrations in house crickets (P = 0.690).

**Figure 3:**
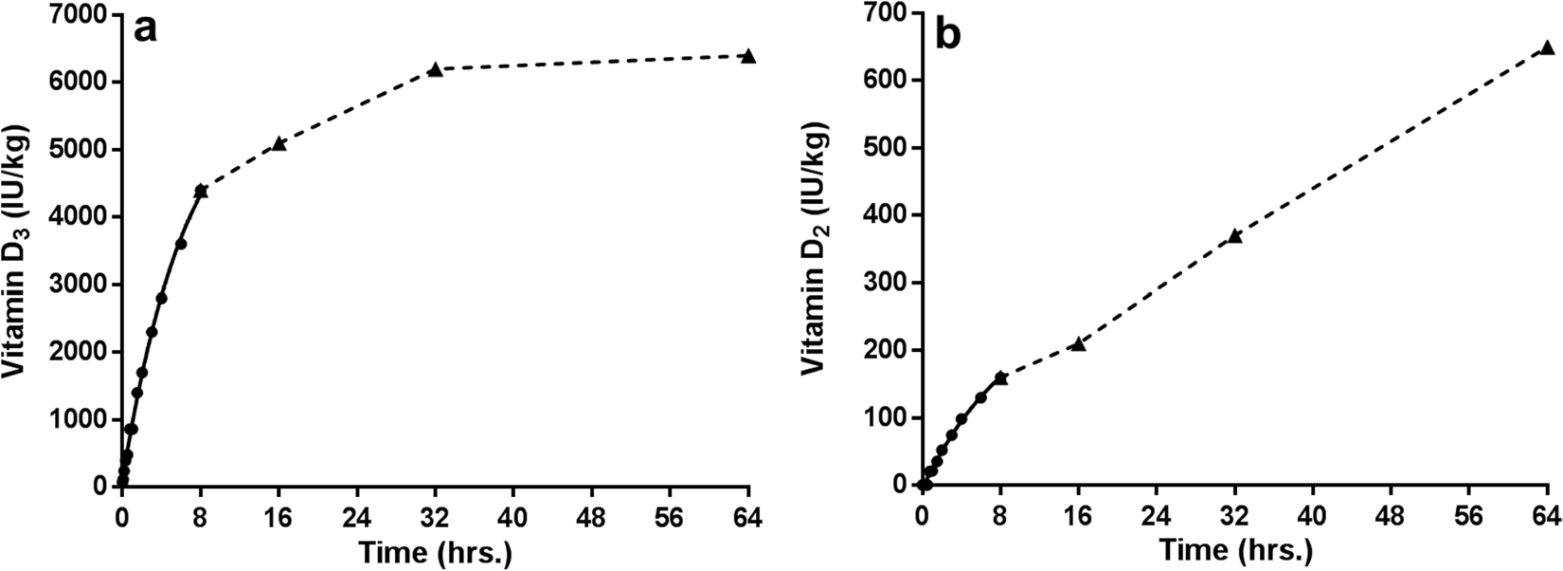
Vitamin D_3_ (**a**) and D_2_ (**b**) concentrations (IU/kg of fresh matter) in yellow mealworm larvae exposed to UVb radiation for varying amounts of time. Circles indicate continuous UVb exposure, whereas triangles indicate UVb exposure for 8 hours per day.

In the third experiment a clear relationship between the duration of UVb exposure and the synthesis of vitamin D_3_ and D_2_ was found (Fig. 3). Vitamin D_2_ concentrations increased with prolonged UVb exposure within the chosen time frame (up to 64 hours over 8 days) whereas the vitamin D_3_ concentration stabilised after 32 hours. Within this experiment much more vitamin D_3_ than D_2_ was produced (6400 vs. 650 IU/kg).

## Discussion

Our experiments revealed that, similar to vertebrates, some insects are capable of *de novo* synthesis of vitamin D_3_. The above-ground living migratory locusts and house crickets and the substrate dwelling yellow mealworms showed elevated vitamin D_3_ concentrations after exposure to UVb radiation. For the other substrate dwelling species, BSFL, UVb exposure did not elevate vitamin D_3_ concentrations compared to the control, although substantial concentrations were found. Furthermore, vitamin D_2_ concentrations were higher in UVb exposed BSFL in the first two experiments, and in migratory locusts exposed to the higher irradiance UVb (experiment 2). Yellow mealworms attained higher vitamin D_2_ concentrations in both experiments, although the difference was not significant in the second experiment due to a low sample size (n = 3).

The vitamin D_3_ synthesis capacity varied between species. For instance, after the first experiment the vitamin D_3_ levels in house crickets were an order of magnitude greater than the levels found in migratory locusts. This is despite the fact that locusts were exposed to higher levels of UVb (Table 3) and unlike house crickets did not have access to a shelter (egg crates) from the UVb radiation. However, when locusts were exposed to the higher UVb irradiance in the second experiment their vitamin D_3_ levels were approximately double those found in the house crickets. The reasons for this discrepancy are unknown but there might be a species-specific UVb threshold to start vitamin D formation, which could be a function of the location of the vitamin D precursor in the insects’ exoskeleton and the UVb filtering properties of their exoskeleton. Such adaptations would be functional for coping with the amount of UVb radiation encountered under natural circumstances; migratory locusts are a diurnal desert-dwelling species, whereas house crickets are crepuscular. Similarly, vitamin D_3_ synthesising capacity has been described to differ between nocturnal and diurnal gecko species^27^ and skin pigmentation in humans affects vitamin D_3_ production^28^. The conversion of pre-vitamin D_3_ to vitamin D_3_ depends on temperature^6,12^. However, the lower temperature of the unexposed insects compared to UVb exposed insects (~1 °C) would only decrease the conversion rate by ~2%^29^.

**Figure 4:**
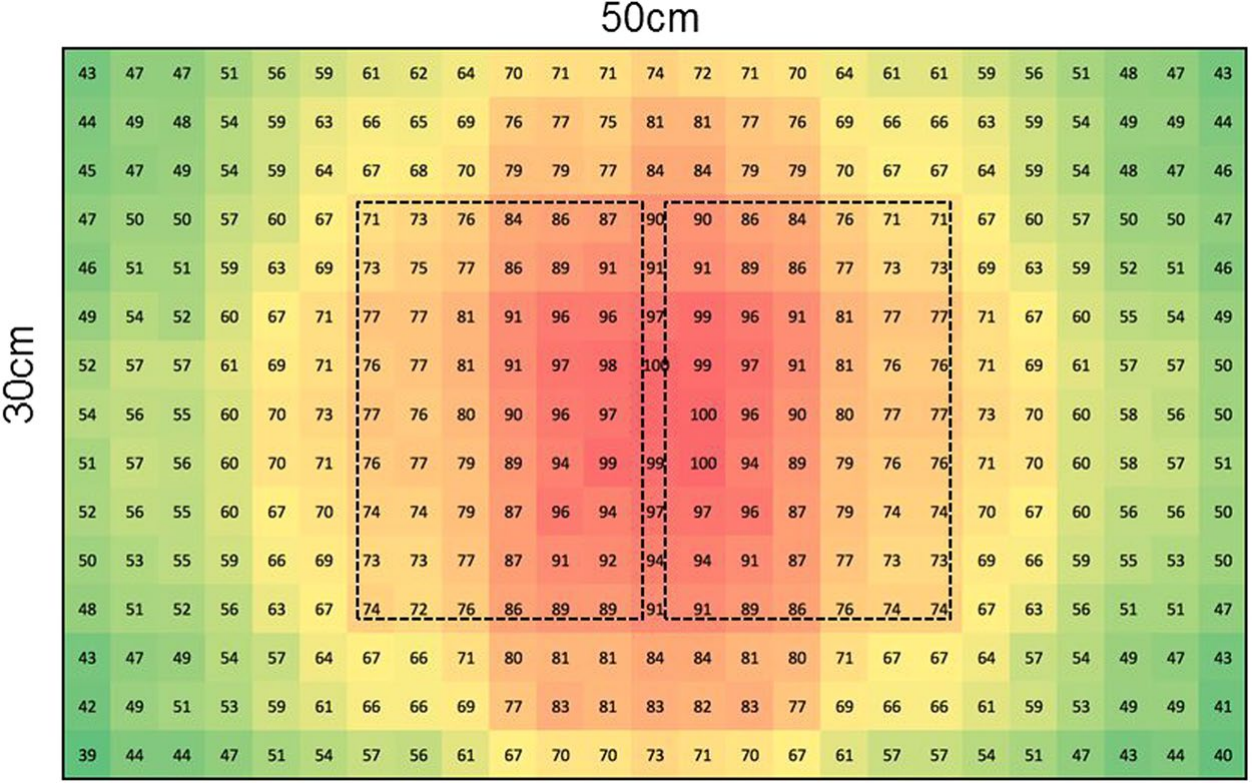
The spread of UVb irradiance as a percentage of the maximum measured at a distance of 11 cm from the light source. Dotted rectangles depict the containers used in the third experiment.

Under our experimental conditions the UVb exposed yellow mealworms had far higher vitamin D_3_ concentrations than the other species tested. As there was a large difference in vitamin D_3_ concentrations between exposed and unexposed yellow mealworms these levels were due to UVb exposure. This species is negatively phototactic; it hides from light which would minimize UVb exposure and consequent vitamin D formation under natural conditions^30^. However, in these experiments the larvae could only hide under each other and a thin layer of food and were therefore exposed to relatively high levels of UVb compared to natural circumstances. As recorded in Table 4, the maximum irradiance directly under the lamp averaged UVI 3.3. This moderate level of irradiation is within the range provided by natural sunlight and although un-natural for this species, did not have any observed negative effects upon the larvae^31^. Exposure to the lamp with the higher irradiance UVb lamp (experiment 2) resulted in higher vitamin D_3_ concentration than exposure to the lower irradiance UVb lamp (experiment 1). The yellow mealworms exposed for 8 hours or more (experiment 3) had a higher vitamin D_3_ concentration than those in the second experiment, although the latter were exposed to the same lamp for a longer period of time. This may be explained by two factors; firstly the layer of mealworms was thicker in the second (~2.0 cm) than in the third experiment (~0.5 cm) resulting in a larger proportion of the mealworms being continuously exposed in the latter experiment. Secondly, the UVb irradiance distribution differed between the second and third experiment due to the utilization of larger containers in the second (50 × 30 cm; Fig. 4, large rectangle) than in the third experiment (11 × 16 cm Fig. 4, small rectangles). Hence, the mealworms in the third experiment were effectively exposed to a higher UVb irradiance than in the second experiment. If the mealworms in the third experiment were equally distributed within their container without overlapping each other, they were on average subjected to a UVb dose of 1.26 J/cm^2^/day. The UVb dose encountered under natural circumstance depends on many variables including location on earth, time of day, time of year, and amount of cloud cover^32^. The UVb dose for the US, averaged over the year, is between 1.2 and 4.1 J/cm^2^/day which indicates that the dosage used in this experiment are comparable to UVb dosages that can be encountered in exposed locations under natural circumstances although these dosages would be considerably higher than this negatively phototactic species would receive in its native microhabitat^33,34^.

In both the second and third experiment mealworm vitamin D_3_ concentrations are far higher than values published for insects to date (Table 1). However, data from wild caught blister beetles (*Tegrodera aloga*) collected in Arizona during late spring show even higher values (8,370–11,280 IU/kg fresh insect; Mark Finke, Personal observation). These concentrations were similar to those found in tuna, salmon and mackerel, renowned for having a high vitamin D_3_ content^28,35,36^ which is believed to be acquired dietarily via several accumulation steps in the food chain^36^.

BSFL are known as efficient converters of organic waste, including waste colonised by fungi^37,38^. The moistened chicken feed provided to the BSFL prior and during the first two experiments is prone to fungal colonization at temperatures between 26 and 31 °C. The three-day old BSFL had high starting levels of vitamin D_2_ and D_3_ (Table 2). They were provided with chicken feed with a high vitamin D_3_ concentration (Table 2) during their first three days of life. Furthermore, they were illuminated by an array of fluorescent tubes (TLD18W840NG, Phillips, Eindhoven, the Netherlands), which emits a small fraction of UV light^39^. After exposure to UVb these fungi could have formed a dietary source of vitamin D_2_ for the BSFL. However, feed samples were not tested at the end of the experiments to ascertain fungal contamination or vitamin D_2_ content. At the end of the two experiments the vitamin D_3_ levels in both the UVb exposed and unexposed BSFL remained high. Vitamin D_2_ levels also remained high in exposed larvae, but were below the limit of detection in the unexposed BSFL at the end of the experiment. During larval development, BSFL grow 4000 times their starting weight^40^, thereby greatly diluting their initial vitamin D_2_ levels if deprived of UV light. Dietary vitamin D_3_ concentrations of the BSFL feed differed greatly (12,000 vs. 580 IU/kg) between the first and second experiment. However, larval vitamin D_3_ concentrations were similar in both experiments. Why the vitamin D_2_ levels were higher in UVb exposed BSFL, but the vitamin D_3_ levels were similar in the BSFL remains to be investigated further. In vertebrates, vitamin D_2_ and D_3_ can be absorbed and metabolised differently due to differences in their binding affinity and their hydroxylation rate^41^. Vitamin D_2_ is for instance less potent than vitamin D_3_ in humans, monkeys and birds, whereas it is more potent in rats^41^. An alternative explanation for the difference in vitamin D_2_ concentrations in the BSFL is that this species synthesizes vitamin D_2_ instead of vitamin D_3_. Typically, plants, yeasts and fungi synthesize vitamin D_2_ and animals synthesize vitamin D_3_, although exceptions to this general rule are known in the plant kingdom^12,13,42,43^. These plants synthesize vitamin D_3_ and metabolise it to 25(OH)D_3_ and 1,25(OH)_2_D_3_^42,43^. In certain plants vitamin D promotes differentiation and elongation of roots, possibly via the stimulation of calcium uptake^42,44,45^. Whether vitamin D fulfils a function in invertebrates is not well understood^13,46^.

It has been suggested that vitamin D_3_ is an inactive end product^13^. However, its sterol precursor 7DHC is well studied as a precursor of insect moulting hormones (ecdysteroids)^47–49^. Unlike vertebrates, insects cannot synthesize sterols and therefore depend on either a dietary source or on symbionts, capable of *de novo* synthesis of sterols, to form these ecdysteroids, which are essential to attain normal growth, development and reproduction^47–50^. In most insect species 7DHC is a minor sterol component, however, in yellow mealworms it comprises 17% of all sterols^51^. Figure 3a shows that the vitamin D_3_ concentration in yellow mealworms reached a plateau in the third experiment. This could be because no more vitamin D_3_ was formed because the precursor 7DHC was limiting, or that vitamin D_3_ was still being formed and metabolised at the same rate. Alternatively, it might suggest that a quasi-equilibrium between 7DHC and its photoproducts had been reached, as occurs in vertebrate skin to prevent overproduction of previtamin D, or that photodegradation of formed vitamin D was taking place^6,52^. Future studies should determine which vitamin D metabolites are formed and whether these metabolites have a function in insects.

## Conclusions

This study indicates that: 1) migratory locusts, house crickets and yellow mealworms can synthesise vitamin D_3_ *de novo* after UVb exposure, but attain different concentrations, 2) higher vitamin D levels can be attained with exposure to higher UVb intensities, and 3) vitamin D_3_ levels in yellow mealworms increase until a maximum concentration is reached during prolonged UVb exposure.

## Methods

Three experiments were conducted. In the first experiment, four insect species were exposed to a lamp which emitted a low UVb irradiance. In the second experiment, these four species were exposed to a lamp which emitted a higher UVb irradiance. In the third experiment, one insect species was exposed to the same UVb lamp as in the second experiment, but for different durations.

### Animal housing and feeding

In the first two experiments migratory locusts (*Locusta migratoria* (L.); Orthoptera: Acrididae), house crickets (*Acheta domesticus* (L.); Orthoptera: Gryllidae), yellow mealworms (*Tenebrio molitor* (L.); Coleoptera: Tenebrionidae), and black soldier fly larvae (BSFL; *Hermetia illucens* (L.); Stratiomyidae: Diptera) were used. The first two species were provided by a commercial insect rearing company (Kreca, Ermelo, the Netherlands) and the latter two species were obtained from colonies maintained at the Laboratory of Entomology, Wageningen University, Wageningen, the Netherlands. In both these experiments the insects were subjected to one of two treatments; UVb exposed or UVb unexposed. In the third experiment only yellow mealworms were used.

All species were housed in enclosures proven suitable in prior investigations and provided with amounts of feed that allowed *ad libitum* feeding throughout the study^53,54^. Due to differences in housing and nutrient requirements, both the enclosures and provided diets differed between species.

#### Migratory locusts

Seventy five penultimate instar nymphs were housed in glass cages, designed for locust rearing, measuring either 60 × 30 × 35 cm (L × W × H; three per treatment) or 50 × 40 × 35 cm (two per treatment). Five replicates were used per treatment. Heat was provided by a lamp (Model 100 W, Philips, Eindhoven, the Netherlands), eight hours per day. Each enclosure was provided with 50 g fresh perennial ryegrass (*Lolium perenne*) from Unifarm (Wageningen University, Wageningen, the Netherlands) in the morning and with 75 g in the afternoon. Grass that had dried out was removed. In addition, each day the locusts were provided with 15 g of wheat bran (Arie Blok, Woerden, the Netherlands) and 30 g carrots (*Daucus carota*) from a local supermarket. The locusts developed large wings indicating adulthood after 21days in the first experiment and after 33 days in the second experiment. They were then manually taken from their cages.

#### House crickets

Fifty g of fourth instar nymphs were housed in a plastic enclosure (Clear Box XL, Bahag AG, Mannheim, Germany; 50 × 35 × 40 cm) in which egg trays were placed to provide sufficient surfaces for resting. Five replicates were used per treatment. In the first experiment the crickets were provided with a chicken feed diet (Opfokmeel farmfood; Agruniek Rijnvallei Voer B.V., Wageningen, the Netherlands) whereas in the second experiment a commercial cricket feed (Kreca, Ermelo, the Netherlands) was used. Furthermore, in both experiments crickets were provided with 30 g carrots each day obtained from a local supermarket. Drinking water was provided via a water dispenser (Gebroeders de Boon, Gorinchem, the Netherlands) with a piece of paper tissue placed in the opening to prevent drowning. In both experiments most crickets reached the adult stage as indicated by the formation of large wings, after 31 days. They were then taken from their cages by hand.

#### Yellow mealworms

Twenty five g of larvae with a length of 3–5 mm were housed in a plastic crate measuring 50 × 30 × 10 cm. Five replicates per treatment were used in the first experiment, and three replicates were used in the second experiment. All were provided *ad libitum* with commercial mealworm feed (Kreca, Ermelo, the Netherlands) and provided daily with 100 g carrots from a local supermarket. When the first pupa was seen in a container, 21 days after the start of the first and 29 days after the start of the second experiment, all mealworms were separated from their substrate with a 1 mm sieve.

#### Black soldier flies

One hundred g of three day old larvae were housed in lidless plastic enclosures (Faunarium type pt2665, Hagen, Holm, Germany) measuring 36 × 23 × 23 cm. Five replicates were used per treatment. In the first experiment the larvae were provided with a chicken feed diet (Opfokmeel farmfood; Agruniek Rijnvallei Voer B.V., Wageningen, the Netherlands), whereas in the second experiment the larvae were provided with commercial cricket feed (Kreca, Ermelo, the Netherlands). Once per day 80 g of dry feed was mixed with 200 ml of tap water and added to each container. In both experiments the first BSF reached the pre-pupal stage after seven days, as shown by the darkening of their integument. All BSF were then taken from their container rinsed with tap water to remove adhering feed residues, and subsequently dried on paper towels.

For all species each replicate batch of insects was then equally divided into subsamples by means of a two-way sample splitter (Model RT 25, Retsch, Haan, Germany) and stored in closed containers at −20 °C until further processing and subsequent analysis.

### UVb exposure

Half of the specimens for each of the four species in experiment 1 and 2 were allocated to a treatment exposed to UVb, whereas the other half were allocated to a control treatment not exposed to UVb. Ultraviolet light was provided by means of 23 W fluorescent compact lamps (Model D3^+^ 10% UVb, Arcadia, Surrey, UK). The lamps used in all three experiments had a similar spectral power distribution across visible wavelengths, but the UV irradiance was higher in the lamps used in the second and third experiment (Fig. 1a and b). All lamps were placed vertically in the middle of each enclosure and were switched on for 8 hours per day. The minimum distance between the UV lamps and the insects differed between species due to differences in cage dimensions; for the migratory locusts and BSFL this was 8.0 cm, for the house crickets it was 10.0 cm and 11.0 cm for the yellow mealworms.

In the control treatments, a long drinking glass (15 × 7.5 cm Blokker BV, Amsterdam, the Netherlands) was placed over the compact lamp which filtered out its UVb output (Fig. 1a). The outside of all enclosures were covered with dark plastic to prevent stray light, including UVb, from entering. During the experiments two UV meters were used, one to determine UVb irradiance (Solarmeter model 6.2, Solartech Inc., Harrison Township, MI, USA) and one to determine the UV index (Solarmeter model 6.5, Solartech Inc., Harrison Township, MI, USA).

For the migratory locusts, house crickets and yellow mealworms, the UV compact lamps were randomly rotated between the experimental treatment cages every three days, to correct for potential differences in UVb output between individual lamps. For the BSFL, which have a shorter life cycle, lamps were rotated daily. In both the first and second experiment the lamps were on for 8 hours per day.

In the third experiment the influence of UVb exposure time on vitamin D content was determined. For this, 30.0 g of yellow mealworm larvae, approximately 10 mm long, was placed in each of sixteen plastic containers (16 × 11 × 6 cm). Ten grams of mealworm feed and 10 g of carrots were added to each container at the beginning of the experiment and, for the treatments lasting longer than two days the same amount was added every two days. The yellow mealworms were then exposed for 0, 5, 10, 20, 30, 45, 60, 90 120, 180, 240, 360, 480, 960, 1920 or 3840 min to the high irradiance compact lamps at a distance of 11.0 cm. Animals in the latter three treatments were UVb exposed for 8 hours per day, and therefore harvested after 2, 4 or 8 days of exposure. One sample was analysed per time point.

### Spectral and temperature measurements

UVb irradiance and UV index of all lamps were determined every five days. Measurements were taken perpendicular to the lamps at the minimum distance between the insects and the lamps (See Animal housing and feeding). Ambient room temperature was set at 24 °C and monitored by means of a memory thermometer (TFA Dostmann GmbH, Reicholzheim, Germany). Enclosure temperatures during the day were measured by means of an infrared thermometer (Model DTG380, Reptile Technologies, Gorinchem, the Netherlands).

Emission spectra of both the low and the high irradiance lamps, with and without the control glass filter in place, were obtained using an Ocean Optics USB2000+ spectral radiometer with a UVb compatible fibre-optic probe with cosine adaptor (Ocean Optics Inc., Dunedin, Florida USA). Spectrometer recordings were made at a distance of 10 cm from the lamp surface.

### Chemical analyses

At the start of the experiments samples were taken from all four species and their feed, and analysed *in duplo* for vitamin D_2_ and D_3_. Insect samples stemming from each replicate in the three experiments were analysed *in simplo* for vitamin D_2_ and D_3_. All insect samples were immersed in liquid nitrogen, homogenised with an analytical mill (Model 11a, IKA, Wilmington, USA) and analysed at TNO Triskelion (Zeist, the Netherlands).

Vitamin D_2_ and D_3_ concentrations in the feed were determined in accordance with Official Method of Analysis (AOAC) 2002.05. In short, after the addition of an internal standard (vitamin D_2_) and basic hydrolysis, vitamin D_3_ was extracted with di-isopropyl ether. The fraction that contains vitamin D_2_/D_3_ was separated by preparative normal-phase liquid chromatography (LC). After evaporation and dilution in methanol-water, vitamin D_3_ was determined by reversed-phase LC with UV detection at 265 nm. A separate test portion was analysed in parallel to confirm the absence of endogenous vitamin D_2_.

Vitamin D_2_ and D_3_ in the insect samples was determined in accordance with AOAC 2011.12. In short, an internal standard solution with deuterated vitamin D_2_ and vitamin D_3_ was added to a ground, homogenised sample. The sample was hydrolysed with ethanolic potassium hydroxide and vitamin D was extracted with di-isopropyl ether. The extract was evaporated, re-dissolved in methanol and vitamin D_2_ and D_3_ were analysed simultaneously using UPLC with tandem mass-spectrometry. The two methods above provide information on total vitamin D concentrations without distinguishing between their esterified and non-esterified forms. The presence of tachysterol in UVb exposed samples was investigated in accordance with Official Method of Analysis (AOAC) 2002.05. No tachysterol was detected, and peak purity of vitamin D was confirmed.

### Statistical analyses

All data was analysed for significant differences (P ≤ 0.05) with SPSS 24.0 (IBM Corporation, Armonk, NY, USA). Temperature and UV data were analysed with a paired sample t-test. Vitamin D data which were normally distributed and had equal variances were analysed via an independent sample t-test, else a Mann-Whitney U test was used. If vitamin D concentrations were below the limit of detection, this detection limit was used for statistical analysis.

### Data availability

The datasets generated during and/or analysed during the current study are available from the corresponding author on reasonable request.
